# Identification and Characterization of a Dominant Sulfolane-Degrading *Rhodoferax sp*. via Stable Isotope Probing Combined with Metagenomics

**DOI:** 10.1038/s41598-019-40000-2

**Published:** 2019-02-28

**Authors:** Christopher Paul Kasanke, R. Eric Collins, Mary Beth Leigh

**Affiliations:** 1 Institute of Arctic Biology, University of Alaska Fairbanks, Fairbanks, USA; 2 College of Fisheries and Ocean Sciences, University of Alaska Fairbanks, Fairbanks, USA

## Abstract

Sulfolane is an industrial solvent and emerging organic contaminant affecting groundwater around the world, but little is known about microbes capable of biodegrading sulfolane or the pathways involved. We combined DNA-based stable isotope probing (SIP) with genome-resolved metagenomics to identify microorganisms associated with sulfolane biodegradation in a contaminated subarctic aquifer. In addition to 16S rRNA gene amplicon sequencing, we performed shotgun metagenomics on the ^13^C-labeled DNA to obtain functional and taxonomic information about the active sulfolane-degrading community. We identified the primary sulfolane degrader, comprising ~85% of the labeled community in the amplicon sequencing dataset, as closely related to *Rhodoferax ferrireducens* strain T118. We obtained a 99.8%-complete metagenome-assembled genome for this strain, allowing us to identify putative pathways of sulfolane biodegradation. Although the 4S dibenzothiophene desulfurization pathway has been proposed as an analog for sulfolane biodegradation, we found only a subset of the required genes, suggesting a novel pathway specific to sulfolane. DszA, the enzyme likely responsible for opening the sulfolane ring structure, was encoded on both the chromosome and a plasmid. This study demonstrates the power of integrating DNA-SIP with metagenomics to characterize emerging organic contaminant degraders without culture bias and expands the known taxonomic distribution of sulfolane biodegradation.

## Introduction

Sulfolane is an anthropogenic organo-sulfur molecule used in some oil and natural gas refineries, resulting in contamination of groundwater at industrial sites around the world^1–3^, including in North Pole, Alaska, where it has contaminated hundreds of private drinking water wells. Despite its emerging importance as a groundwater contaminant, little is known about the environmental fate of sulfolane. Sulfolane biodegradation potential exists in activated sludge, contaminated aquifer substrate, and pristine soil, but the identity of the microorganism(s) responsible remains largely unknown^4–6^. Three sulfolane degraders have been previously identified through pure-culture-based studies, with mixed enrichment cultures reportedly degrading sulfolane more quickly than pure cultures^7–9^. It remains unknown how diverse, widespread, or abundant sulfolane degraders are in the environment, particularly in contaminated aquifers, where this information is valuable in assessing plume longevity and identifying remediation strategies, including natural attenuation and accelerated bioremediation.

Molecular techniques like stable isotope probing (SIP) are powerful tools for examining the active members from environmental microbial communities involved in the biodegradation of emerging organic contaminants such as sulfolane. ^13^C-based SIP is the process of exposing a microbial community to a chemical compound highly enriched in ^13^C, which otherwise accounts for roughly 1% of all carbon. The microorganisms that metabolize the ^13^C-labeled substrate will incorporate the heavy isotope into their biomolecules^10^. Analyzing the ^13^C-enriched DNA enables the identification of functionally relevant community members through DNA sequencing approaches including 16S rRNA gene amplicon and shotgun-metagenomic sequencing. Shotgun metagenomic sequencing can also shed light on the functional capabilities of the active organisms and identify metabolic pathways potentially being utilized^11,12^.

To date, the environmental microorganisms known to be capable of degrading sulfolane to date have all been isolated from temperate regions. They include a *Shinella* sp. from Okinawa Japan, a *Variovorax* sp. from Alberta Canada, and a *Pseudomonas* sp. from Illinois USA^7–9^. To our knowledge, the identity of sulfolane degrading microbes in a subarctic aquifer, like that found in North Pole, Alaska, have not previously been reported. We performed DNA-SIP with ^13^C-labeled sulfolane in contaminated North Pole, Alaska, aquifer substrate to elucidate the identity of subarctic sulfolane degraders while circumventing culture bias. We combined DNA-SIP with shotgun metagenomics to taxonomically identify microbes involved in sulfolane degradation as well as to gain insights into their genetic potential and possible degradation pathways that may be used to process sulfolane. This study demonstrates that, by combining these techniques, it is possible to not only identify but also obtain high-quality draft genomes of unknown emerging contaminant degraders from environmental samples.

## Results

### Isolation of ^13^C-labeled DNA

**Figure 1:**
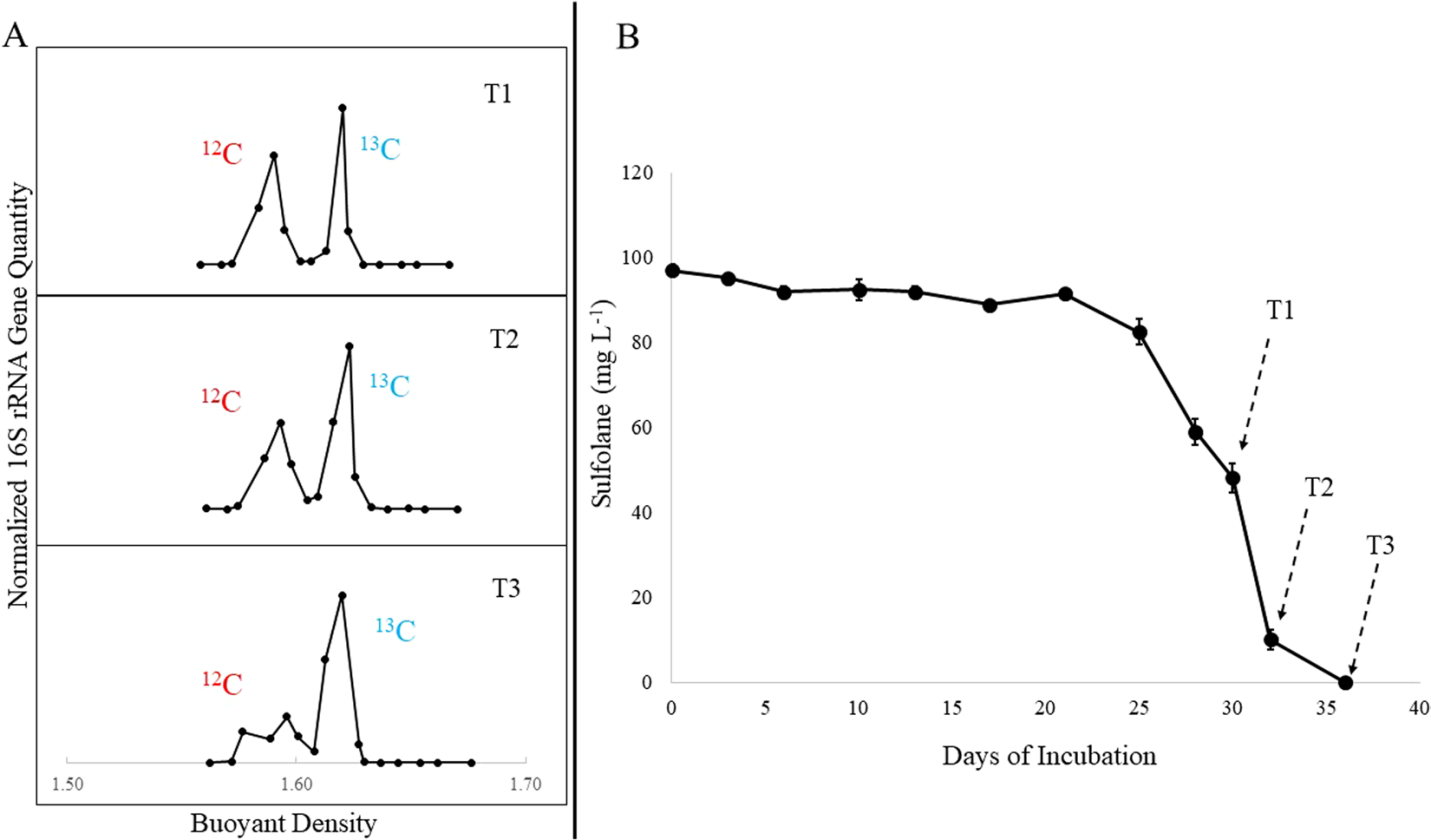
(**A**) Quantitative PCR results showing relative abundance of 16S rRNA gene copies in density gradient fractions after separation of the labeled and unlabeled DNA in representative microcosms amended with ^13^C-labeled sulfolane. The ^13^C-labeled fractions increase in relative abundance over time demonstrating the incorporation of ^13^C into prokaryotic DNA as sulfolane is biodegraded. (**B**) Sulfolane loss over time in SIP microcosms. Dashed arrows indicate when triplicate microcosms were destructively harvested. Error bars represent standard deviation.

Quantitive PCR results showed a clear separation between the heavy and light DNA in the density gradient (Fig. 1A). In addition, there was an increase in the relative abundance of labeled DNA over the course of the incubation in the microcosms amended with labeled sulfolane. That indicated the labeled carbon was being assimilated by members of the microbial community. As expected, there was no quantifiable heavy (^13^C-labeled) DNA in any of the control microcosms amended with ^12^C-sulfolane (Supplementary Dataset 1).

### Microbial Community Analysis

Statistical analyses showed that the microbial communities in the SIP “heavy” fractions, SIP “light” fractions, and ^12^C-sulfolane controls were all significantly different from each other (MRPP, p < 0.05). These groups were also significantly different from the time-zero total community samples with the largest difference between T0 and the ^13^C-labeled communities (A = 0.66, p ≪ 0.05). However, once a community shift occurred and sulfolane degradation was initiated, which occurred by day 28, there was no significant change in the microbial community profile over the course of the incubation within the SIP “heavy” fractions, SIP “light” fractions, and ^12^C-sulfolane controls. Since there was no difference between T1, T2, and T3 communities (Fig. 1B), we combined the three timepoints from each treatment group into one representative sample for community analysis.

**Figure 2:**
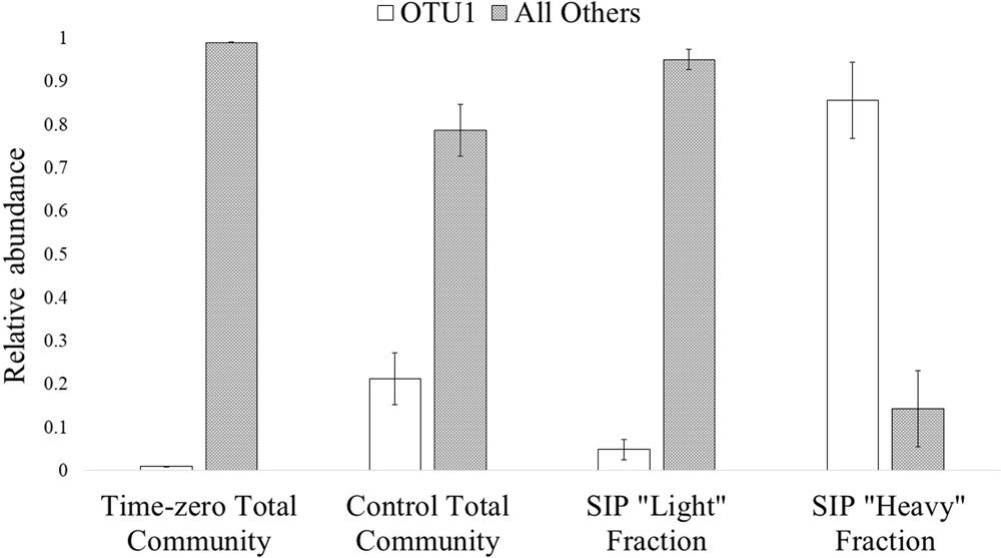
Abundance of OTU1 in all 16S rRNA gene amplicon community types. Since no differences were found between community structure over the course of incubation, all replicates from each timepoint (28, 32, and 36 days) were averaged together for the control (n = 7), unlabeled fraction (n = 9), and labeled fraction (n = 9) communities. Time zero represents the subarctic aquifer substrate prior to sulfolane exposure (n = 3). “Control” refers to the community incubated with unlabeled sulfolane. Error bars represent standard deviation from the mean.

The microbial communities associated with the ^13^C-labeled DNA fractions were very low in richness (Chao1 65.6 ± 27.8) and diversity (Inverse Simpson index 1.4 ± 0.3) with only one OTU (OTU1) comprising 85.7 ± 8.7% of the total labeled microbial communities (Fig. 2). A BLAST comparison of the 253-bp partial 16S rRNA gene sequence for OTU1 showed this gene fragment was 99% identical to five different species from three genera of the *Comamonadaceae* family (*Rhodoferax ferrireducens*, *Rhodoferax saidenbachensis*, *Limnohabitans parvus*, *Acidovorax facilis* and *Acidovorax radicis*). Analysis of the full 16S rRNA gene obtained via shotgun sequencing uniquely identified OTU1 as a *Rhodoferax* sp., as detailed below. The next two most abundant community members were *Lysobacter* sp. and *Bacteriovorax* sp., which, when combined, comprised less than 3% of the total labeled community (1.3 ± 2.0% and 1.1 ± 0.9% respectively). The dominant phylotype in the unlabeled or “light” fractions of the ^13^C-sulfolane incubation was OTU15 from the *Sphingomonadaceae* family, which represented 7.4% of the unlabeled community. OTU1 comprised 4.9% of the light fraction’s total community.

Although OTU1 (presumably *Rhodoferax* sp.) was uniquely dominant in the ^13^C-labeled microbial community, it was not the only dominant organism detected in the total community analysis of the control microcosms that were exposed to ^12^C-sulfolane. In the ^12^C-control cultures, OTU1 was co-dominant with OTU3 (identified as *Arthrobacter* sp.), which represented 23.2 ± 3.4% of the total microbial community, while OTU1 constituted 21.3 ± 6.0%. OTU3 was also significantly more abundant than OTU1 in the starting community (paired t-test, df = 2, t = −8.28, p = 0.014). Even though OTU3 was a dominant member of the total community in ^12^C-sulfolane control cultures, it was ruled out as a sulfolane degrader due to its lack of incorporation of ^13^C from sulfolane into DNA.

As expected, the microbial community showed more OTU richness and diversity prior to sulfolane exposure (Chao1 2209.0 ± 89.1, Inverse Simpson 140.2 ± 12.7). The dominant ^13^C-enriched OTU1 was the 18^th^ most abundant phylotype in the T0 total community and comprised less than 0.1% of the microbial community prior to sulfolane exposure and incubation. The two most abundant T0 total community phylotypes were identified as *Geobacte*r *spp*. and represented 4.2% and 3% of the total community, respectively. These OTUs were not found in the SIP “heavy” community. Although there was a small subset of archaea in the T0 microbial community, no labeled archaeal DNA was detected in the sequence data.

### Metagenomic analyses

By shotgun sequencing the low-diversity heavy SIP fractions we were able to obtain a high-quality draft metagenome assembled genome (MAG) of the putative sulfolane-degrading microorganism. A total of 2.9 M paired-end 2 × 250 bp reads (1.5 Gbp) remained after quality control and trimming, which produced an assembly containing 12,437 nodes and a total length of 14 Mbp. A single large connected component made up 49% of the total size (6.9 Mbp) with a mean sequencing depth of 53x (Supplementary Fig. 1). This component was composed of 395 contigs with an N50 of 146 kbp and a longest contig of 614 kbp. An additional 262 kbp were contained in 41 contigs on 5 additional connected components, of which two were closed circular plasmids and one was complete but not circular (Supplementary Fig. 2). The average length of the 11,998 unconnected contigs was 572 bp; the mean depth of these contigs was 0.7x. The 395 contigs from the large connected component were defined as a MAG and annotated in JGI-IMG/ER. A total of 6537 protein coding genes were identified; the GC content was 60.84%. CheckM analysis found the genome to be 99.8% complete with only 0.50% contamination (defined as redundancy of putative single-copy genes).

**Figure 3:**
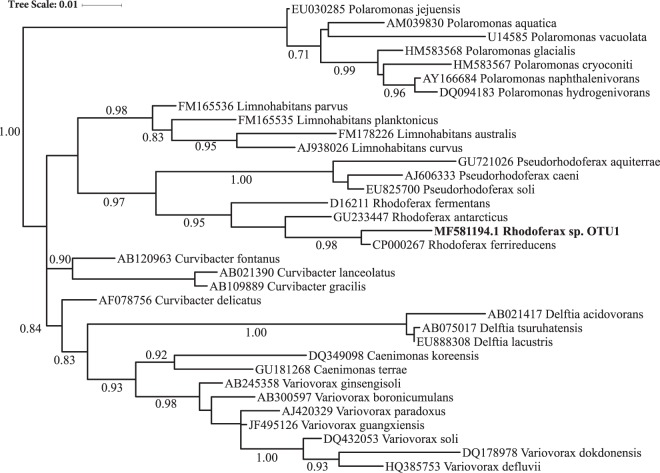
Phylogenetic tree of *Rhodoferax sp*. OTU1 compared to closest type strain bacteria. Tree is based on full-length 16S rRNA gene sequence similarity. SH-aLRT node confidence values ≥ 0.70 are shown.

The 16S rRNA gene for the MAG was aligned to the representative sequence of OTU1, the sulfolane-degrading *Comamonadacaea* sp. identified in the community dataset, using BLAST^13^. The 16S rRNA gene assembled from the metagenome was 100% identical to the amplicon, matching 253 of 253 bases and identifying this draft genome as the genome of the labeled sulfolane degrader. BLAST comparisons of the full 16S rRNA gene sequence from the metagenome-assembled genome identified the sulfolane degrader as a *Rhodoferax* sp., being 99% identical to *Rhodoferax ferrireducens* strain T118. A phylogenetic tree based on the 16S rRNA gene is also provided (Fig. 3).

It has been suggested that sulfolane biodegradation may follow the 4S-pathway described for diobenzothiophene desulfurization^8^. This pathway employs the genes *dszA*, *dszB*, and *dszC* to oxidize the sulfur to sulfite, which abiotically oxidizes to sulfate in aerobic conditions. We searched the MAG for these genes that may be involved with sulfolane degradation. A protein BLAST search found a gene that is homologous to *dszA* (42% identical, 59% positive, e-value of 10^−109^) in the genome and a second, distantly related homolog (Supplementary Fig. 3) repeated 5 times on an associated plasmid (Supplementary Fig. 4). The MAG does not contain *dszB*, which cleaves the sulfite moiety to complete the remineralization of sulfur in the 4S-desulfurization pathway^14,15^. However, the genome of the *Rhodoferax* sp. does have 84 genes involved with sulfur metabolism, including complete sulfur oxidation (*sox*) and alkanesulfonate utilization (*ssu*) pathways (Supplementary Fig. 5). To our knowledge there has only been one other gene, identified using *Escherichia coli* mutants, proposed to be associated with sulfolane metabolism^16,17^. This gene is known as *thdF* and the MAG of the sulfolane assimilating *Rhodoferax* sp. does not contain this gene.

## Discussion

**Figure 4:**
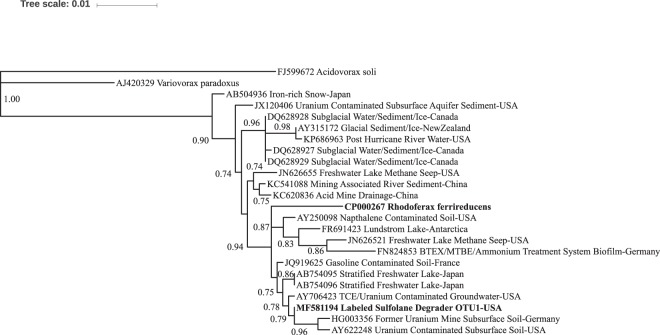
Phylogenetic tree of *Rhodoferax sp*. OTU1 and closest related non-type strain bacteria based on full-length 16S rRNA gene similarity. All non-type sequences represented here are >99% similar to *Rhodoferax sp*. OTU1. SH-aLRT node confidence values ≥ 0.70 are shown. *Rhodoferax ferrireducens* is in bold for reference.

Using DNA-SIP combined with metagenomics, we have identified a single OTU as the primary sulfolane-degrading organism in subarctic aquifer substrate and provided DNA evidence that strongly suggests it is a member of the *Rhodoferax* genus (Fig. 3). Of the 178 OTUs detected in the ^13^C-labled SIP fractions, OTU1 was identified as the dominant microorganism incorporating carbon from sulfolane in this subarctic aquifer substrate. Although initial analysis of the 253-bp amplicon data revealed OTU1 as a member of the *Comamonadaceae* family, the full 16S rRNA gene provided more a more rigorous genus-level taxonomic resolution. Phylogenetic analysis showed that OTU1 was 99% similar to type strain *Rhodoferax ferrireducens* T118 (Fig. 3), which was isolated from subsurface sediments collected in Oyster Bay, VA, USA. This relationship was also supported by whole genome database comparisons. When non-type strains were included in the phylogenetic analysis, most of the closest relatives were found in contaminated soil or groundwater with the top 20 originating from freshwater or terrestrial environments (Fig. 4). Although this is the first report of a *Rhodoferax sp*. degrading sulfolane, it is not surprising since members of the *Rhodoferax* genus are commonly found in contaminated freshwater environments and have been implicated in the degradation of other contaminants including herbicides, naphthalene, and benzene^18–20^.

Prior to identifying *Rhodoferax* sp. OTU1 as a sulfolane degrader, the only environmental microorganisms known to degrade sulfolane originated from subtropical, humid subtropical, or continental climates. In Western Canada a sulfolane degrader was isolated from a contaminated aquifer and identified as a *Variovorax* sp. which is in the same family (*Comamonadaceae*) as *Rhodoferax* sp. OTU1^8^. Two other environmental isolates have been reported to degrade sulfolane. *Pseudomonas maltophilia* was isolated from the soil of an abandoned strip mine near Cambria, Illinois; USA and grew on sulfolane as the sole carbon source^7^. A novel *Shinella* sp. was isolated from soil in the Yambaru area of Okinawa Main Island; Japan and grew on sulfolane as the sole sulfur source^9^. To our knowledge, the only other report of a sulfolane-degrading bacterial species was a mutated strain of *Escherichia coli*, which was not isolated from the environment but did yield insights into sulfone degradation pathways^16^. A mutation in the *thdA* gene allowed this *E*. *coli* strain to degrade sulfolane via a novel sulfone oxidase enzyme. The authors proposed *thdA* to be a regulator gene for several genes involved in the metabolism of organo-sulfur compounds, including *thdF* thiophene oxidase^16^. The novel sulfone oxidase was never identified and *thdA* has not been sequenced, but the genetic sequence for *thdF* has been published^17^. We did not find *thdF* homologs in the MAG of the degrader, but that does not rule out the unidentified sulfone oxidase as being involved in sulfolane metabolism.

**Figure 5:**
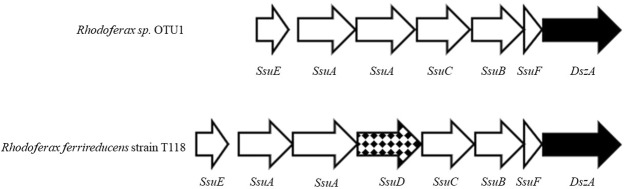
Order of alkanesulfonate metabolism genes in *Rhodoferax sp*. OTU1 and *Rhodoferax ferrireducens* strain T118. Open arrows indicate shared ssu genes. The checkered arrow is the monooxygenase *ssuD* which is present in this operon in strain T118 but not the sulfolane assimilating species. However, a homolog to *SsuD* is elsewhere in the genome of the sulfolane-assimilating *Rhodoferax* sp. The *dszA* homolog is present in both species at the end of the *ssu* operon.

**Figure 6:**
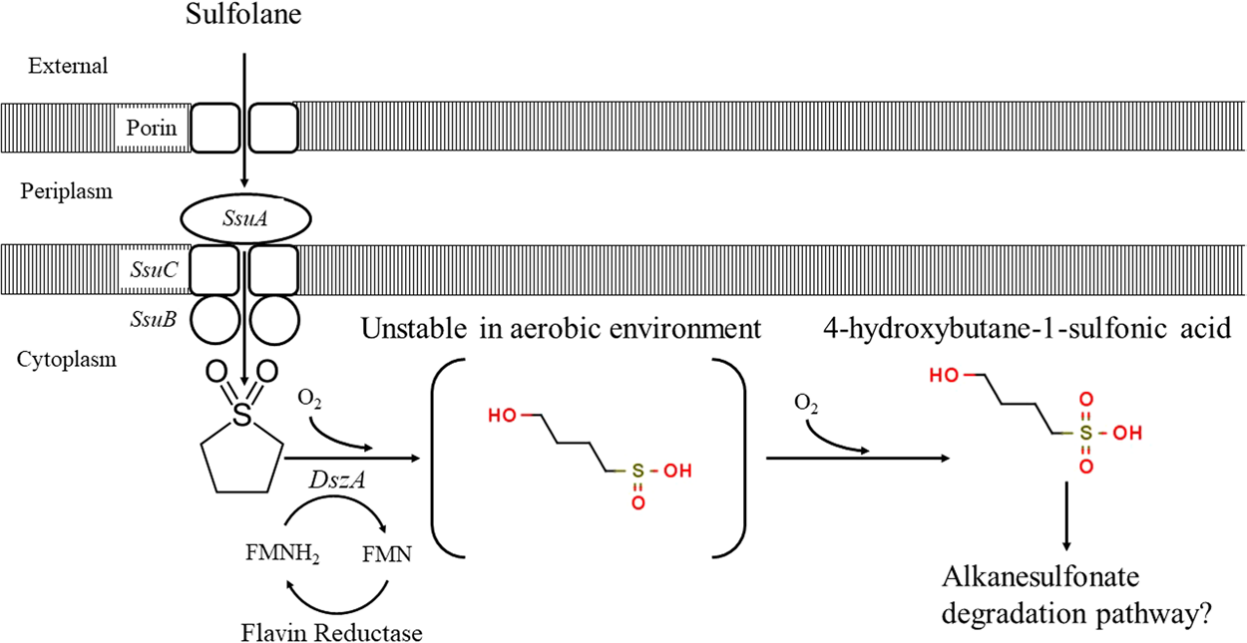
Schematic of proposed sulfolane biodegradation pathway. Protein identifications and Kegg Orthology values are as follows: Sulfonate transport system substrate-binding protein (*SsuA*; K15553), Sulfonate transport system ATP-binding protein (*SsuB*; K15555), Sulfonate transport system permease protein (*SsuC*; K15554), Dibenzothiophene sulfone monooxygenase (DszA; K22220). The flavin reductase refers to either *SsuE* (flavin reductase; K00299) or *DszD* (Genbank Accession AB051429.1; no KO or E.C. values available), which have coding regions in close proximity to *dszA* on the chromosome and plasmid respectively.

It has been proposed that the biodegradation of sulfolane followed the 4S-desulfurization pathway for dibenzothiophene due to structural relatedness of the compounds and the production of sulfate as a mineralization product^8^. This pathway involves the use of the *dsz* operon involving genes *dszA*, *dszB*, and *dszC*^21^. The gene product of *dszA* opens the ring structure after dibenzothiophene is converted to dibenzothiophene-5,5-dioxide by *dszC*^15^. We found no evidence of *dszB* being present in the MAG for the degrader, suggesting that sulfolane biodegradation in this strain does not utilize the 4S-desulfurization pathway. However, the MAG does encode a homolog to *dszA* at the end of an alkanesulfonate utilization pathway (Fig. 5). In addition, 5 copies of another *dszA* homolog are present on an IncP-family plasmid in the metagenomic co-assembly (Supplementary Figs 2–4). This plasmid has a copy number of about 3 relative to the chromosome, suggesting that the plasmid-borne *dszA* homolog is present at 15 times the copy number of the genomic *dszA* homolog. This is perhaps not surprising considering that the dibenzothiophene pathway is also generally plasmid-borne (Denis-Larose *et al*. 1997, Denome *et al*. 1994). If either of these DszA homologs can act on sulfolane as DszA does to dibenzothiophene, the remaining compound would be 4-hydroxy-butane sulfinic acid. Under aerobic conditions sulfinic acids can oxidize to sulfonic acids, with aliphatic sulfinic acids being more reactive than aromatic ones^22^. Although speculative, it is feasible that the resulting product (4-hydroxy-butane-sulfonic acid) is then degraded in a similar fashion to other alkanesulfonates and/or taurine, which this species is genetically equipped to process (Fig. 6, Supplementary Fig. 5).

Greene *et al*.^8^ previously found that mixed cultures are more efficient at degrading sulfolane than isolates, but it was unclear if this was due to the exchange of specific nutrients between organisms, the removal of growth-inhibiting products, the combined metabolic attack on the substrate, or some combination. We were surprised to find no compelling evidence of sulfolane assimilation in any species other than *Rhodoferax* sp. OTU1 throughout the course of this labeling study. Although there were other labeled microbes in this community, they were in extremely low abundance and were likely labeled due to the scavenging of labeled biomolecules^23^. In addition, the ^13^C-labeled community structure did not change substantially over the entire SIP incubation period, which might be expected if sulfolane metabolites were being degraded in series. Although *Rhodoferax* sp. OTU1 represented ~25% of the community in the control (unlabeled sulfolane) microcosms, it was co-dominant with an *Arthrobacter* sp. Despite the abundance of the *Arthrobacter sp*. in the controls, we found no labeled *Arthrobacter* DNA, which strongly suggests it is not involved in sulfolane biodegradation. It is likely this species is an opportunistic, heterotrophic, soil-bacterium that can tolerate the experimental sulfolane concentrations allowing it to become co-dominant with the *Rhodoferax* sp. However, if the *Arthrobacte*r sp. was degrading sulfolane as a method of detoxification or energy production and not assimilating the carbon, we would not identify it as a degrader using these methods. We are currently working to isolate *Rhodoferax* sp. OTU1 into pure culture in order to enable definitive characterization of the sulfolane degradation pathway.

## Conclusion

We have identified a *Rhodoferax* sp. as being the dominant and likely the exclusive sulfolane-degrading microorganism in enrichment cultures from contaminated subarctic aquifer substrate. By combining ^13^C-DNA-SIP with 16S rRNA gene amplicon sequencing and shotgun metagenomics, we were able to not only resolve the taxonomy of this degrader, but also gain insights into how it may be metabolizing sulfolane. We also suggest that sulfolane degradation by this *Rhodoferax* strain does not proceed following the previously proposed model^8^. Although an *Arthrobacter* sp. comprised ~25% of the total community in the ^12^C-sulfolane controls during active sulfolane biodegradation, it was not assimilating sulfolane carbon into its DNA and therefore likely not involved in the biodegradation process. We caution that simply analyzing changes in the microbial community profile during sulfolane biodegradation studies is not sufficient to determine species function and may be misleading. Although this study cannot confirm that *Rhodoferax* sp. OTU1 actively degrades sulfolane within the contaminated aquifer, it enables further studies of biodegradation potential *in situ*, such as through characterizing the environmental distribution of this organism and identifying environmental factors driving its abundance.

## Methods

Subsurface samples used as inoculum for SIP studies were collected from Flint Hills Resources property located in North Pole, Alaska (64.7511°N, 147.3519°W) where groundwater sulfolane levels ranged from 0 to 34.8 mg L^−1^ at the time of sample collection^24^. The sediment and groundwater samples used in this study came from a sulfolane-contaminated subarctic aquifer and a detailed site characterization and history of sulfolane use can be found in Kasanke and Leigh^3^. The aquifer sediment used in this study consisted of augured material from the installation of a new monitoring well at depths between 3 and 9 m below ground surface. All sediment was sieved through a 2-mm screen prior to use. Groundwater used in this study came from an existing monitoring well approximately 30 m from where sediment was collected. The well was screened 18.25 m below the ground surface and has stable historical sulfolane concentrations of approximately 125 µg L^−1^ ^24^. Groundwater was collected in September 2012 and sediment in March 2013. Both were stored at 4 °C (the approximate average aquifer temperature) until use in February 2016. The top of the water table at time of sampling was 3 m below ground surface and the aquifer has an average temperature of 3.4 °C^24^.

### Stable Isotope Probing

SIP microcosms each contained 12.5 g of soil and 40 ml of groundwater combined in a 160-ml serum bottle. Since previous sulfolane biodegradation studies showed more predictable degradation curves when nutrients were added, 5 ml of a 1X Bushnell-Haas mineral nutrient solution was added to each microcosm (final concentration: magnesium sulfate 0.022 g L^−1^, calcium chloride 0.0022 g L^−1^, monopotassium phosphate 0.11 g L^−1^, diammonium hydrogen phosphate 0.11 g L^−1^, potassium nitrate 0.011 g L^−1^, ferric chloride 0.006 g L^−1^). To examine the composition of the initial microbial community, three of these microcosms were immediately harvested and stored at −80 °C for DNA extraction and 16S rRNA gene sequencing. The remaining microcosms were assigned to two treatment groups containing nine microcosms each. One group was amended with a sterile aqueous solution of custom-synthesized ^13^C-labeled sulfolane (99% ^13^C, Microbial Insights, TN) and the other was amended with commercially available (predominantly ^12^C) sulfolane (Acros Organics, Belgium) as a control. The target starting sulfolane concentration in all microcosms was 100 mg L^−1^. All microcosms were incubated under aerobic conditions at 4 °C. Aliquots (1 ml) of the liquid phase were periodically sampled from each microcosm for sulfolane quantification using gas chromatography-mass spectrometry^3^. Once significant sulfolane loss was detected (39.15% removed at 28 days of incubation), three microcosms from each treatment group were destructively harvested for microbial community characterization (Fig. 1B). The remaining microcosms were harvested in the same manner for two additional timepoints (days 32 and 36 of incubation) when additional sulfolane loss was observed (89.64% and 100% respectively) (Fig. 1B).

### DNA extraction

To obtain enough bacterial DNA from these low-biomass samples for density gradient separation (>1 µg), DNA was extracted using a modified version of the MO BIO PowerSoil DNA isolation kit (MO BIO Laboratories #1288-100). For each DNA extraction, 0.5 g of microcosm sediment and 100 µl of microcosm supernatant was combined into a single PowerSoil Bead Tube, with five DNA extractions performed for every harvested microcosm. Extractions were performed following the MO BIO PowerSoil protocol except that DNA was concentrated by combining all five extracts from a microcosm onto a single PowerSoil Spin Filter and eluted into a single collection tube using 100 µl (2 × 50 µl) of C6 elution buffer. Double-stranded DNA was quantified using a Qubit fluorometer. All DNA extracts contained between 1.84 and 4.8 µg total DNA.

### Separation and detection of ^13^C-DNA

^13^C-labeled DNA was separated from the unlabeled DNA via isopycnic centrifugation in cesium trifluoroacetate (CsTFA) following a previously described protocol^25^. Briefly, between 1.8 and 3 ng of total DNA was added to 5 ml of a CsTFA solution (Amersham 17-0847-02) diluted to a buoyant density of ~1.62 g ml^−1^. Density gradients were created by ultracentrifugation in a Beckman Coulter Optima L-100 XP ultracentrifuge using the fixed-angle Beckman NVT 100 rotor at 45,600 r.p.m. and 25 °C for 72 hours. The gradients were divided into 20 fractions (buoyant density 1.28–1.82) and qPCR targeting the bacterial and archaeal 16S rRNA gene was performed on each fraction^26^. The normalized abundance values for targeted genes were calculated by dividing the abundance of each fraction by that of the most abundant fraction within the same gradient. Fractions containing heavy DNA (labeled) were pooled together and those containing light DNA (unlabeled) were pooled together (Fig. 1B) within each individual gradient. The primarily ^12^C-sulfolane controls were also fractionated and pooled similarly to control for any DNA contamination in heavy fractions. Pooled fractions from all samples were then subjected to 16S rRNA gene sequencing as described below.

### 16S rRNA gene sequencing

The V4 region of the bacterial and archaeal 16S rRNA gene was amplified using Illumina fusion primers as described by Caporaso *et al*.^27^. PCR output for all samples was normalized using a Life Technologies SequalPrep Normalization plate. The normalized products were pooled. After Ampure clean up, QC and quantitation the pool was loaded on a 500-cycle reagent cartridge (v2) Illumina MiSeq flow cell and sequenced in paired end 2 × 250 bp format using custom V4 sequencing and index primers^27^. Base calling was done by Illumina Real Time Analysis (RTA) v1.18.54 and output of RTA demultiplexed and converted to FastQ with Illumina Bcl2fastq v1.8.4.

FASTQ files were analyzed using mothur software (1.35.1) following a modified version of the standard MiSeq SOP (accessed March 2016)^28,29^ as described by Martinez *et al*.^26^. Briefly, all sequences had a quality score of 25 or greater and the maximum contig length was set to 275. All unique sequences were aligned against the SILVA SEED v119 database and chimera checking was performed using the mothur implementation of UCHIME^29,30^. Unique operational taxonomic units (OTUs) were defined at a level of 97% sequence similarity and taxonomy was assigned using SILVA SEED v119 taxonomy database^30^. To account for differences in sequence coverage, the number of sequences was subsampled to the number of sequences in the least covered sample (8142). Differences between microbial communities were assessed using nonparametric Multi-Response Permutation Procedures (MRPP) from a Bray-Curtis distance matrix^31^. All statistical analyses were performed with R statistical software using the vegan package^32^.

### Metagenomic sequencing

Shotgun metagenomic sequencing was performed on one ^13^C-labeled DNA extract from each of the sampling timepoints. The DNA was prepared for sequencing using a Nextera XT DNA Library Prep Kit and sequencing was performed on an Illumina MiSeq using a standard v3 flow cell and paired end 2 × 300 bp sequencing format with an average insert size of 275 bp. Raw reads were trimmed and quality filtered using bbduk in the bbmap package (https://jgi.doe.gov/data-and-tools/bbtools/bb-tools-user-guide/). After trimming and filtering, raw sequences were error corrected and assembled using SPAdes version 3.10.1^33^. The de Bruijn graph assembly was visualized with Bandage^34^, which indicated the presence of a large connected component containing a single metagenome assembled genome (MAG) and several complete plasmids (Supplementary Fig. 2). As a check, contigs were also binned by tetranucleotide frequency using VizBin^35^ into a single MAG. For further processing we used the large connected component MAG. MAG bin quality was assessed for contamination and completeness using CheckM and the MAG and plasmids were annotated using RAST, PATRIC, and JGI/M ER pipelines^36–39^. Phage and plasmid genes were also identified with PHASTER^40^. The full-length 16S rRNA gene (1541 bp) was extracted from the MAG and queried against Genbank and RDP databases using BLASTN to determine the relationship of this genome to other known microorganisms^41–43^. Using BLASTN, the full-length 16S rRNA gene from the MAG was queried against the representative OTU sequences in the 16S rRNA amplicon dataset to identify the OTU of the genome we obtained^44^. Raw reads from the 16S rRNA amplicon sequencing and shotgun metagenomic sequencing are available in the sequence read archive (SRA) under accession #SRP136637. This Whole Genome Shotgun project has been deposited at DDBJ/ENA/GenBank under the accession QEII00000000. The version described in this paper is version QEII01000000. The MAG assembly and annotation is publicly available in the JGI IMG/ER database under accession # 181102. Sequence assemblies for the plasmids extracted from the MAG are available in the Supplementary Materials.

### Phylogeny

We compared the full-length 16S rRNA gene (1541 bp) of the dominant ^13^C-labeled organism to that of both the type strain and non-type strains of the closest relatives. The 16S rRNA sequences of close relatives were obtained from the RDP and GenBank databases^41,42^. Prior to tree construction the sequences were aligned using the RDP tree builder program and manually checked using Seaview version 4^41,45^. The maximum likelihood phylogenetic tree was constructed using PhyML^46^ in SeaView under the GTR model ^45^after alignment in MUSCLE^47^ and edited online using iTOL^48^. Shimodaira-Hasegawa approximate likelihood ratio test (SH-aLRT) node confidence values were calculated during tree construction^49^.

## Supplementary information

Supplemental Information

Supplementary Dataset 1

Supplementary Dataset 2
