# Supplementary material for: Identification and Characterization of a Dominant Sulfolane-Degrading Rhodoferax sp. via Stable Isotope Probing Combined with Metagenomics
Source: Sci Rep. 2019 Feb 28;9:3121. doi: 10.1038/s41598-019-40000-2 (PMC6395730; doi:10.1038/s41598-019-40000-2)
Supplement: Supplementary file 1 — Supplemental Information [file 41598_2019_40000_MOESM1_ESM.pdf]

Title: Identification and Characterization of a Dominant Sulfolane-Degrading *Rhodoferax sp.* via Stable Isotope Probing Combined with Metagenomics

Running Title: Sulfolane Stable Isotope Probing and Metagenomics

Authors: **Christopher Paul Kasanke**[1*], R. Eric Collins[2], and Mary Beth Leigh[1]

[1] Institute of Arctic Biology, University of Alaska Fairbanks; USA

[2] College of Fisheries and Ocean Sciences, University of Alaska Fairbanks; USA

[*] Corresponding Author

E-mail: cpkasanke@alaska.edu

All authors declare there are no competing interests in relation to the work described.

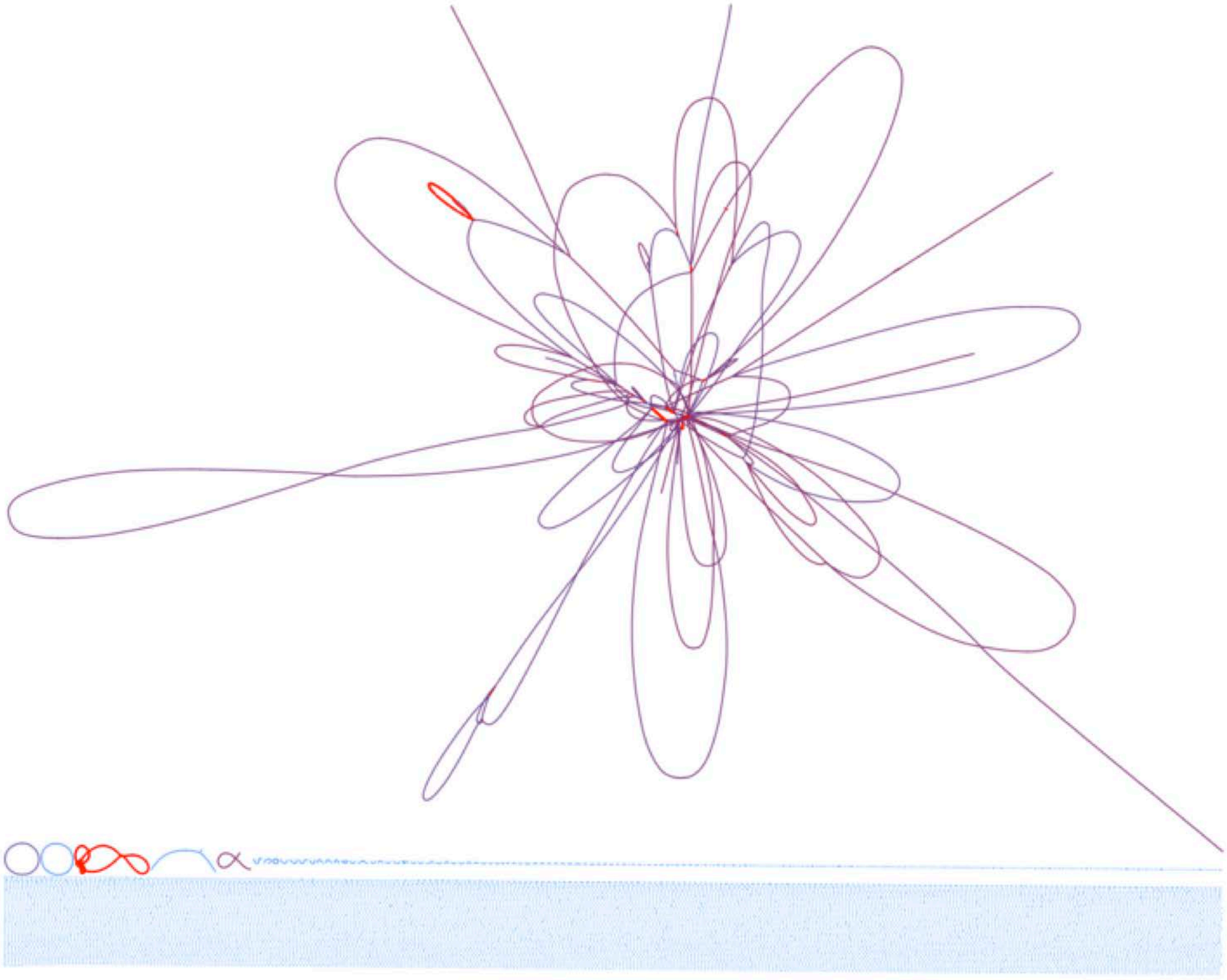

Supplementary Figure 1: Metagenome co-assembly de Bruijn graph from SPAdes visualized with Bandage. Sequencing depth is scaled to color, from blue (0x) to red (100x). The mean depth of the large connected component is 53.1x.

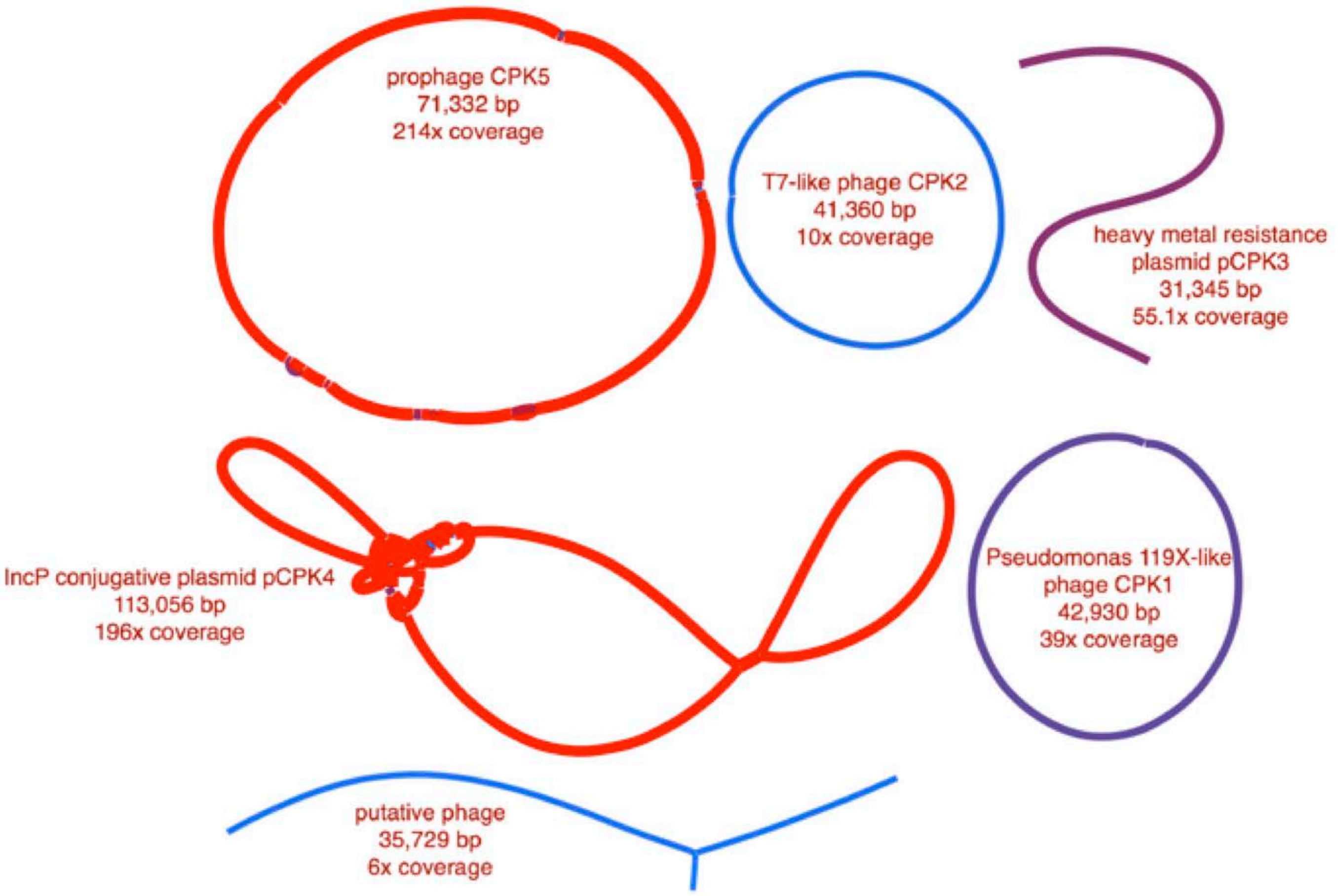

Supplementary Figure 2: Extrachromosomal elements from metagenome co-assembly de Bruijn graph visualized with Bandage. Sequencing depth is scaled to color, from blue (0x) to red (100x). Phage and plasmid genes were identified with PHASTER.

Tree scale: 0.1

Supplementary Figure 3. A maximum likelihood phylogenetic tree of amino acid sequences homologous to *dszA*. Tree was constructed using PhyML in SeaView after alignment in MUSCLE.

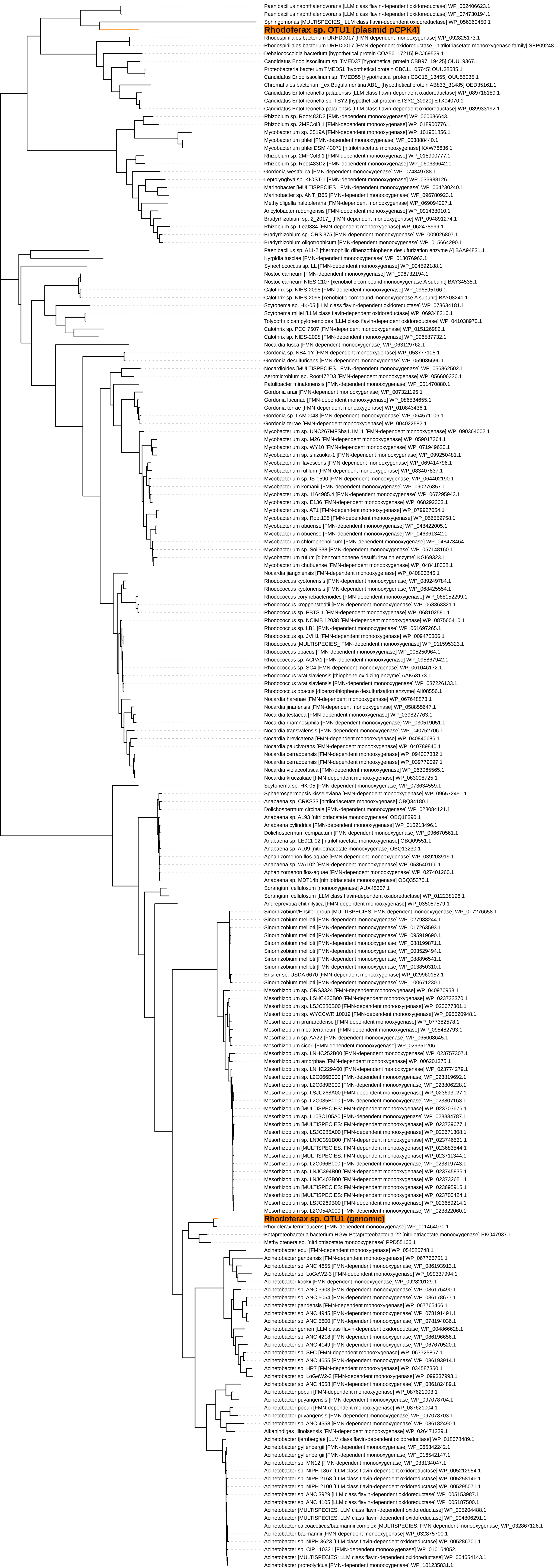

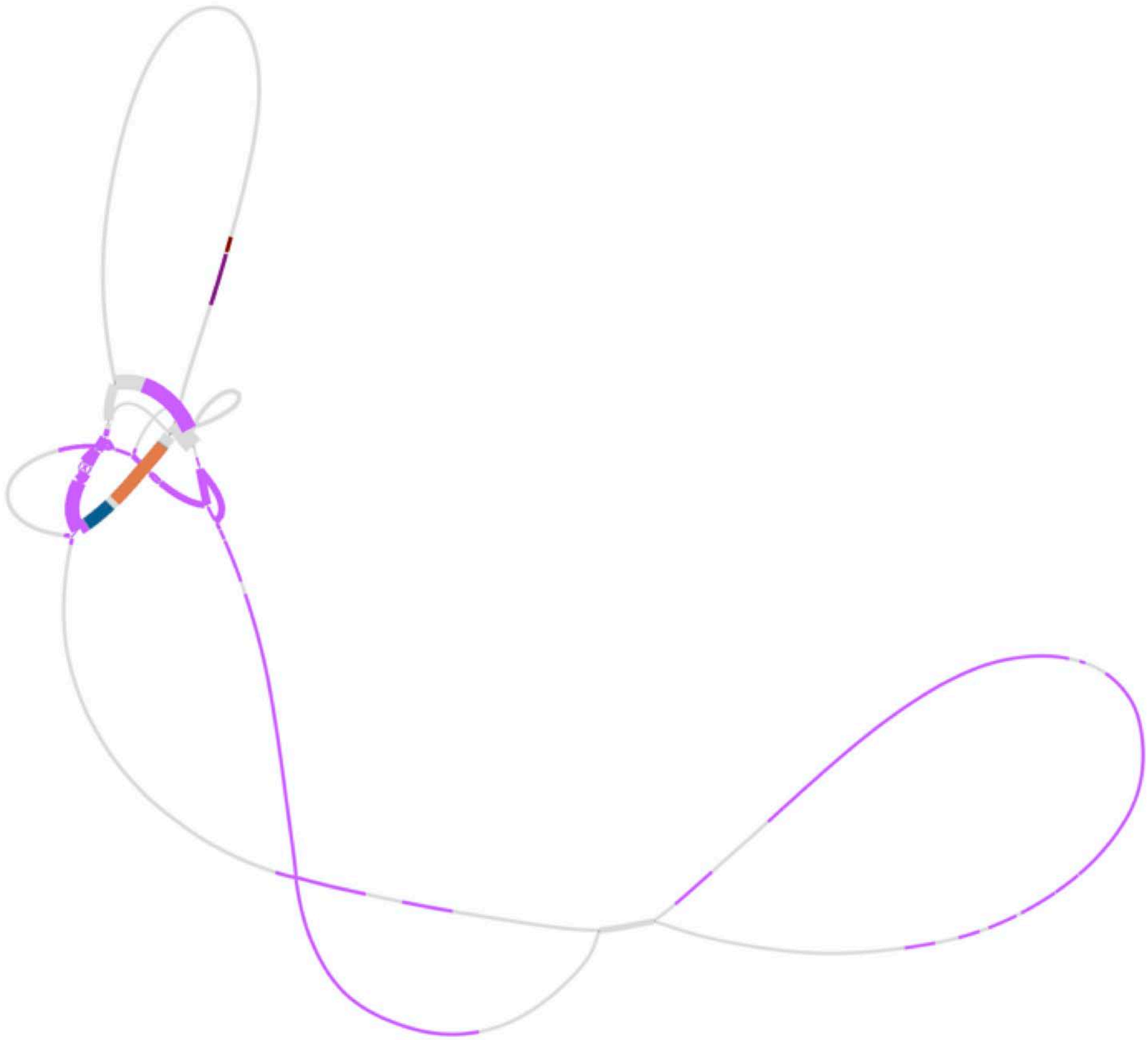

Supplementary Figure 4. IncP conjugative plasmid pCPK4 from the metagenome co-assembly de Bruijn graph visualized with Bandage. Sequencing depth is scaled to node width (max depth 894x). pCPK4 is highly similar (99%) over large regions (>40%; purple) to an IncP plasmid from *Pseudomonas* spp. and other soil microorganisms. The orange segment is a putative flavin-using monooxygenase homologous to *dszA*, which is only distantly related to the chromosomal dszA-like gene. The green segment is a putative flavin reductase homologous to *dszD*. These genes are flanked by transposases.

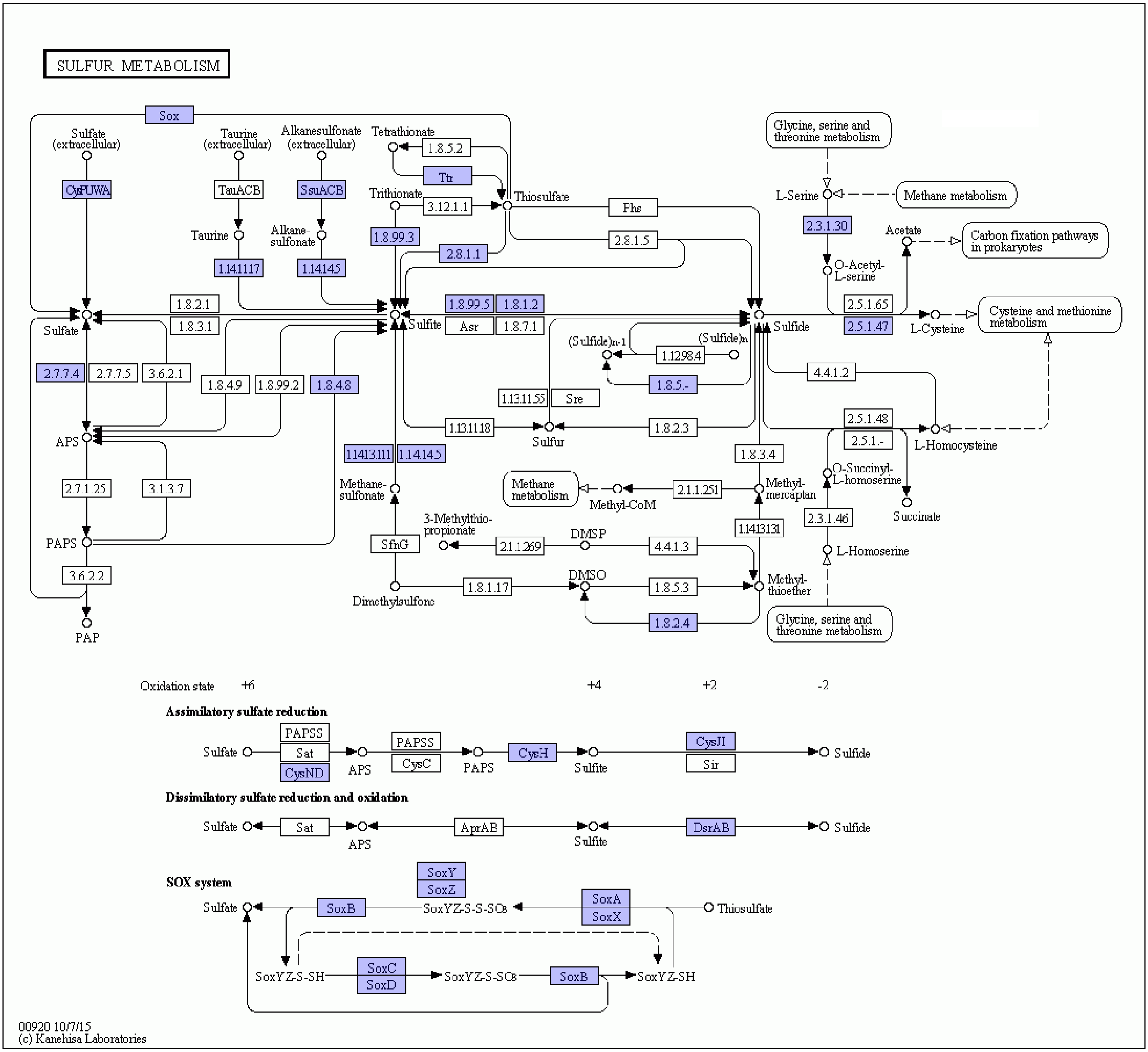

Supplementary Figure 5. Kegg map of the metabolic pathways associated with sulfur metabolism (Kanehisa et al. 2017). Squares in blue are genes that are contained in the Rhodoferax sp. MAG.

Kanehisa, Furumichi, M., Tanabe, M., Sato, Y., and Morishima, K.; KEGG: new perspectives on genomes, pathways, diseases and drugs. Nucleic Acids Res. 45, D353-D361 (2017).
